# An Integrated Cost Model Based on Real Patient Flow: Exploring Surgical Hospitalization

**DOI:** 10.3390/healthcare10081458

**Published:** 2022-08-03

**Authors:** Bruno Barbosa Vieira, Augusto da Cunha Reis, Alan de Paiva Loures, Eliel Carlos Rosa Plácido, Fernanda Ferreira de Sousa

**Affiliations:** 1 Production Engineering Department, Federal Center for Technological Education Celso Suckow da Fonseca-CEFET-RJ, Rio de Janeiro 20271-110, Brazil; augusto.reis@cefet-rj.br; 2 Juiz de Fora Federal University Hospital—HU-UFJF, Juiz de Fora Federal University—UFJF, Juiz de Fora 36036-110, Brazil; alan.loures@ebserh.gov.br (A.d.P.L.); elielcarlos33@yahoo.com.br (E.C.R.P.); ferreiradesousa.fernanda@gmail.com (F.F.d.S.)

**Keywords:** healthcare costs, hospital costs, costs and cost analysis, hospitals, teaching, public hospital

## Abstract

Considering the gap observed in studies on health costs, this article aims to propose a cost calculation model for surgical hospitalization. A systematic literature review using PRISMA was conducted to map cost drivers adopted in similar studies and provide theoretical background. Based on the review, an integrated model considering real patient flow was developed using CHEERS guidelines. The micro-costing top-down method was adopted to develop the cost model allowing a balance between the accuracy of the information and the feasibility of the cost estimate. The proposed model fills two gaps in the literature: the standardization of a cost model and the ability to assess a vast number of different surgery costs in the same hospital. Flexibility stands out as an important advantage of the proposed model, as its application enables evaluation of elective and urgent surgeries of medium and high complexity performed in public and private hospitals. As a limitation, the hospital should have hospital information and cost systems implemented. The proposed cost model can provide important information that can result in better decision making. This becomes more relevant in public health, especially in low- and middle-income countries, which faces a lack of resources and whose positive effects can improve healthcare.

## 1. Introduction

According to the latest data from the World Health Organization (WHO), in 2018, the health systems of its 194 member countries spent USD 8.3 trillion, equivalent to 10% global GDP [1]. Despite the significant amount, there are severe shortcomings in providing healthcare, especially in developing countries [2,3]. According to Shrime et al. [4], these deficits include surgical care, an essential component of healthcare systems. Between 2015 and 2030, the Lancet Commission estimated that 143 million additional surgeries per year are needed in low- and middle-income countries to prevent disabilities and save lives, which means a 46% increase over the total of 313 million surgeries undertaken each year [5].

Brazil had the ninth-largest expenditure in healthcare in 2018 among WHO member countries, totaling USD 177 billion, equivalent to 9.5% of its GDP [6]. The Brazilian public health system (SUS) aims to provide the population with universal and equal access to health services [7,8,9,10]. Among providers, hospitals stand out as the most expensive SUS components [11,12], with surgical admissions representing the highest amounts spent by hospitals [13].

Some studies in Brazil have addressed cost management in healthcare, but economic evaluation is still incipient, including public and teaching hospitals. One explanation is that many institutions do not even have cost systems in place yet [14,15,16,17]. Such studies seek to reference costing methods and how to implement these systems. However, in these analyses, there is little or no cost analysis. Worldwide, several studies have been carried out to identify health costs and develop methods to improve this research, thus contributing to better decision making which is based on more reliable evidence [18,19,20]. However, to date, a systematic review has not been undertaken.

Hospitals are complex and expensive structures [21] and the health decision maker should be supported with the best possible foundation. Economic evaluation in health has been applied to provide this basis and one of its forms, cost analysis, is considered the essential element of all evaluations [22,23]. Cost analysis includes the measurement of used amounts of resources and the allocation of unit values or prices to a set of relevant costs [22,23,24].

Within this context, considering the gap observed in studies on health costs, this paper seeks to answer the question: how to determine resources spent in surgical hospitalizations and how to place monetary values to these resources? To solve this, the main objective of this research was to propose a cost calculation model for surgical hospitalization. To achieve this goal, a systematic literature review was conducted to identify existing models and to map the parameters adopted in similar studies. This review did not identify surgical hospitalization cost models. Thus, this study constitutes a first effort to modeling costs of surgical hospitalization.

Application of the model will make it possible to understand the real cost of surgical hospitalizations, analyzed from the hospital perspective, by comparing them with the remuneration values for the hospital foreseen in the health system and also with other hospitals. The correct understanding of this relationship will more precisely direct management actions related to cost reduction, as well as reviews of remunerated amounts.

As delimitation of the research, a cost study was not conducted. For this reason, values resulting from the application of the model in this research are not presented.

## 2. Materials and Methods

As the first stage of the research, a systematic literature review was carried out to identify possible cost models for surgical hospitalizations, cost drivers, or variables. The PRISMA Statement [25] recommendations were followed. Scopus, Web of Science (WoS), PubMed, and CINAHL/EBSCO databases were used to collect data due to the relevance of the journals indexed in them, as well as the range of subjects. The last update of search results reported below was on 15 July 2022. As this research does not involve humans and animals, the systematic literature review protocol registration was not needed.

The search parameters and the Boolean operators used to search for titles, abstracts, and keywords were: (framework OR model OR recommendation OR algorithm) AND (“inpatient cost”) AND (surg*). The first set of terms served to delimit articles related to models or recommendations, while the second aimed to identify studies that dealt with inpatients’ cost and, finally, the third one was used to select studies that mentioned surgery.

After searching databases, the filter for document type “article/review” was applied. The document filter was intended to include original and literature review articles and to exclude studies that were not peer reviewed. The search resulted in 112 documents in Scopus, 39 documents in WoS, 54 documents in PubMed, and 14 documents in CINAHL being selected.

To realize the screening, eligibility, and inclusion stages from the PRISMA protocol, one author read the records and full articles. Subsequently, another author revised independently to validate the selected studies to review.

From the state of the art mapped in the systematic literature review, the empirical part of modeling began. In this sense a Brazilian public teaching general hospital, composed of 156 beds and divided into surgical, clinical, pediatric, day hospital, and ICU, was chosen as the scenario to develop the cost calculation model. This hospital provides services exclusively to SUS and services a macro-region composed of 94 municipalities and approximately 1.5 million inhabitants, with reimbursement by SUS according to healthcare production.

Empirical data to model development were provided by two computer systems used in the hospital. The first one, a cost management system named APURASUS and provided by Brazilian Ministry of Health, adopts the absorption costing methodology that organizes resource data in cost centers according to the hospital structure. The second one, named AGHU, is a Hospital Information System (HIS) that allows for the identification of main cost drivers related to hospitalizations, such as length of stay (LOS), number of exams, and the distinction between surgical hospitalizations and other types, such as clinical and pediatric. The HIS also provides resource-specific data for supplies and medications spent during each surgery in the operating room, providing direct costs.

The three-step modeling process, consisting of conceptualization, modeling, and solution [26], was followed to achieve the main objective of this research. For the first step, a systematic literature review was used in the theoretical field. In the practical field, documentary research was carried out on the systems used by the hospital to understand the real patient flow.

For the modeling step, this study considered the applicable guidelines for Consolidated Health Economic Evaluation Reporting Standards (CHEERS) [23]. The scope was defined to the health sector component with provider (hospital) perspective [4,24]. The operational model’s solution, the third step of development, is materialized in the proposal presented in the Surgical Hospitalization Cost Model topic.

The micro-costing top-down method was adopted to develop the cost model, using direct costing for medical supplies and medicines spent during surgery and absorption costing for other resources. The micro-costing method was adopted to identify and measure costs as it allowed for a greater level of detail when considering costs of materials, medicines, personnel, and other factors. For the valuation of resources, the top-down method was adopted since the retrospective data were aggregated in cost centers of the different areas of the hospital. The micro-costing top-down combination allowed for the distinction of resources spent for different surgical procedures [27]. This method identifies direct costs (costs directly related to a particular service) based on the average cost of services and materials used for the procedure and indirect costs (costs not directly related to a particular service) by departments (cost centers) without detailing at the patient level [28]. This combination is also adequate because there are specific cost components which make it possible to obtain costs directly (supplies and medicines spent during surgery).

## 3. Results

### 3.1. Systematic Literature Review

**Figure 1:**
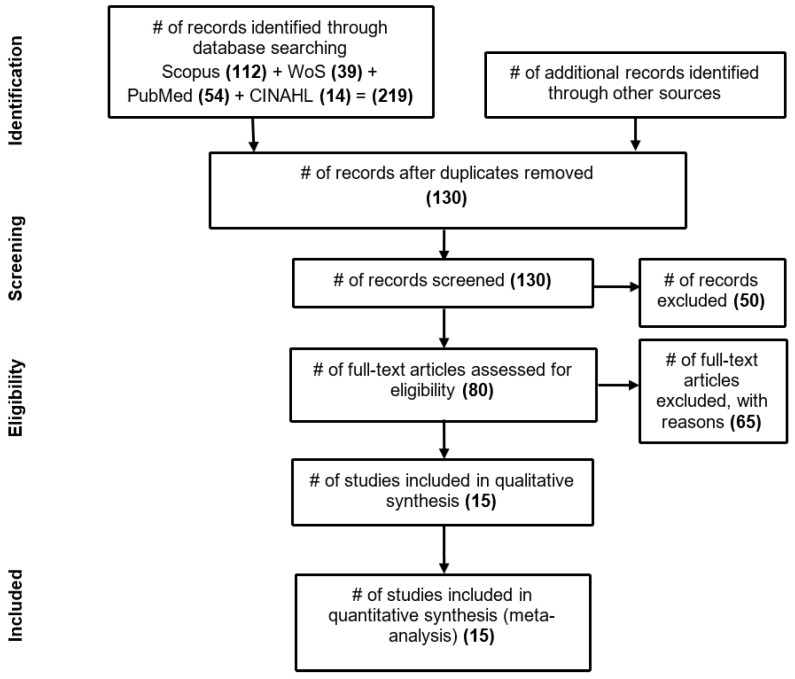
Information flow through the different phases of a systematic review (adapted from the PRISMA Statement [25] (p. 5)).

Figure 1 presents the results obtained in each of the PRISMA Statement flowchart steps.

At the identification stage, databases were consulted according to the search parameters and additional filters already presented. After reviewing the titles of the 219 identified reports, 89 duplicates were eliminated and 130 reports were kept. There was no inclusion of reports identified from other sources.

In the screening stage, the title and summary were read to identify whether the research was related to determining surgical hospitalization costs. In this stage, 50 reports unrelated to the topic were identified and excluded. The reports whose title and summary did not allow for the identification of a relationship were maintained.

For the eligibility stage, 80 full articles would be analyzed. However, four papers were not available for download and were excluded; therefore, 77 articles remained for full reading. This stage aimed to identify the existence of articles that presented a model for calculating the costs of surgical hospitalizations and the cost drivers used. During this stage, 65 articles were excluded due to the reasons presented in Table 1. Finally, 15 articles were selected for inclusion.

Most of excluded studies whose stated objective was to infer surgery costs did not actually carry out the costing process. The 36 records excluded for the main reason (Performs an estimate based on the conversion rate available in a database) make their estimates based on charges made by hospitals, and conversion of these values into costs using a conversion rate (e.g., cost-to-charge ratio). The same reasons why these studies were excluded already was also pointed by Finkler [29] when distinguishing charges and costs.

Therefore, this research differs from the others because the proposed model considers the costs of a hospital-based on its real expenses instead of the cost estimate based on the conversion of amounts charged through a ratio index.

In inclusion stage, 15 articles chosen for eligibility were maintained. Table 2 shows the articles included. Selected studies covered the publication period between 2005 and 2020, with 80% of the studies published within last ten years, revealing that this is a relatively recent topic.

Among the countries in which studies on the subject were conducted, there was a high concentration in the United States, with 11 articles, equivalent to 73% of the total [30,32,33,35,36,37,38,39,42,43,44]. Brazil, Australia, Turkey, and Germany had one study conducted in each country [31,34,40,41].

The studies were published in different journals, with no concentration. Most of the articles analyzed were published in medical journals associated with the specialties whose surgical procedures were related.

Table 3 shows the results of the qualitative analysis of the articles’ content considering the purpose of this literature review to answer the research question. The most relevant result is that no study has proposed a cost calculation model for surgical hospitalizations, revealing that this is a gap in the literature to address this topic which this research comes to fill.

It is also possible to analyze in Table 3 that cost studies are generally applied to a few different surgical procedures. This is due to the complexity of the cost determination process and the methodology used, generally linked to the investigation of the records of each patient.

There were variations in the central objectives of the analyzed studies. However, it is noted that identifying the cost of the procedure was a concern of several researchers. Even so, it is observed that the costing process was carried out differently among the different studies, which can be justified by the absence of a reference model. To fill this gap a surgical hospitalization cost model is proposed in the next topic.

Table 4 shows the types of costs, with information related to cost centers (when used) and resources considered by the respective authors, in each article included in the literature review.

The analysis of Table 4 reveals that there is no established pattern in the studies; however, it is observed that, in most of them, the costs related to personnel, materials, medicines, surgical supplies, laboratory tests, imaging, in addition to maintenance and administrative costs of the ward areas, operating room (surgical center), and intensive care unit, make up the costs of the analyzed surgical procedures.

### 3.2. Patient Flow and Surgical Hospitalization Cost Model

**Figure 2:**
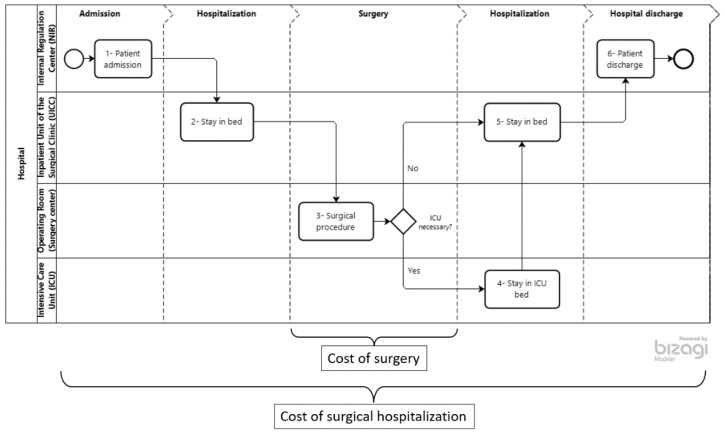
Flow of surgical hospitalization and scope of the study (cost of surgical hospitalization).

From the hospital perspective, the total cost comprises the period from the patient’s admission (preoperative stage) to their discharge (postoperative stage), a process called surgical hospitalization which includes surgery (the main stage) as one of its components. The workflow diagram, created with Bizagi Modeler^®^ software [45] and presented in Figure 2, identifies the stages that make up patient flow in relation to surgical hospitalization compared to the approach that contemplates only surgery.

The proposed model includes three timeframes in the perioperative process which can be seen in Figure 2: preoperative stage, that includes the admission of the patient to the hospital (technical-administrative activities) at the Internal Regulation Center (NIR) and their admission to the Inpatient Unit of the Surgical Clinic (UICC); the operative stage, which includes anesthetic induction, surgery, and anesthetic recovery in the operating room of the surgery center; and the postoperative hospital stage, with a return to the UICC or referral to the Intensive Care Unit (ICU) until the patient’s discharge from the NIR [46].

**Figure 3:**
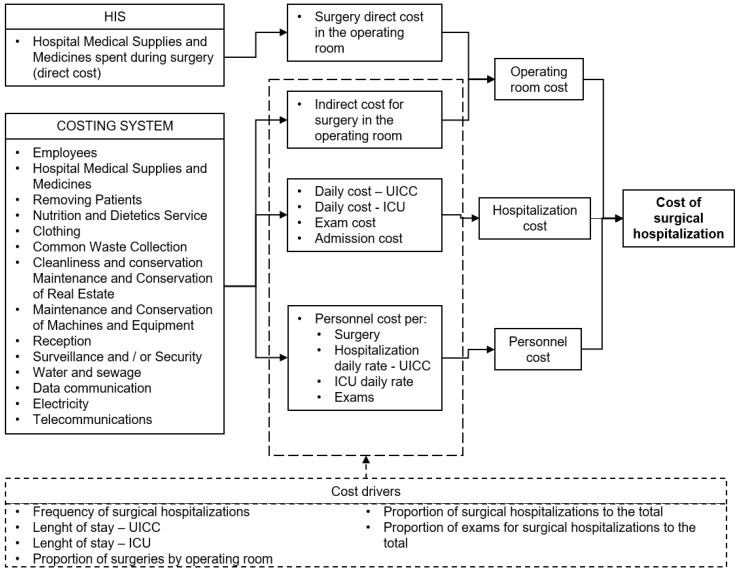
Surgical hospitalization cost model.

Figure 3 presents the model for calculating costs of surgical hospitalizations, in which the “Cost of surgical hospitalization” for each different surgical procedure is the sum of three cost blocks thus organized to facilitate comparisons. The costs related to the preoperative and the postoperative episodes of care are presented as “Hospitalization cost”. Costs related to the operating room, including direct and indirect costs, are presented as “Operating room cost”. This information can be used for comparisons with cost analyses whose scope does not cover the entire patient’s hospitalization, but only the expenses related to the surgery (operative stage). “Personnel costs” are identified separately, as they can be treated independently and thus allow comparability between institutions with different hiring models.

The proposed cost model uses direct costing for medical supplies and medicines spent during surgery and absorption costing for other resources. Absorption costing was used because the studied public hospital adopts the APURASUS system, which organizes data into cost centers, making the extraction and processing of management information more feasible.

The department cost (cost centers) used in the model consists of: Surgical centers (except for materials and medicines already included in the consumption notes); UICC; ICU; Exams (laboratories an imaging exams); and NIR.

The following resources are allocated to the aforementioned cost centers: Personnel; Hospital Medical Supplies and Medicines used in the infirmary; Patient Removal; Nutrition and Dietetics Service; Clothing; Common Waste Collection; Cleanliness and conservation; Maintenance and Conservation of Real Estate; Maintenance and Conservation of Machines and Equipment; Reception; Surveillance and/or Security; Water and sewage; Data communication; Electricity; and Telecommunications.

To apply the model, the number of surgeries and exams, as well as the average LOS at the UICC and ICU, must be identified in a given period. Additionally, the supplies and medicines consumed for each different procedure during the surgery must be identified with their respective values. These data can be obtained electronically from the HIS.

The “Operating room cost” is composed of the sum of the direct unit cost and indirect cost. The direct cost is represented by the median value of Hospital Medical Supplies and Medicines spent during surgery, registered electronically via HIS. The indirect cost for surgery in the operating room is the result of all cost items registered at APURASUS and allocated to the Operating room cost center which is prorated by the respective number of procedures performed in each of the operating rooms from the hospital, except personnel.

The “Hospitalization cost” is obtained through the sum of the daily cost for the UICC cost center, daily cost for the ICU cost center, laboratory and imaging exams performed at respective cost centers, and administrative admission costs, which is called Internal Regulation Center (NIR) cost center in the model. Personnel costs are not included in this block. The daily cost for the UICC and ICU is established by apportioning the values assigned to the cost center by the respective cost driver. Daily costs of these centers are then multiplied by the average LOS specific to each surgical procedure. The exams are prorated as a proportion of the exams requested for surgical hospitalizations concerning the total. The admission costs are prorated as a proportion of surgical hospitalizations in relation to total hospitalizations.

The “Personnel cost” is estimated using the same cost drivers for apportioning the Hospitalization cost in the UICC, ICU, exams, and NIR cost centers, adding the personnel cost for the operating room. To obtain this last information, the proportion of surgeries by each operating room is considered. Personnel cost is calculated by department but segregated from the Operating room cost and the Hospitalization cost.

Table 5 summarizes the relationship explained in the previous paragraphs between the cost components.

The next topic will discuss contributions from the literature to the development of the model.

## 4. Discussion

As presented in Table 3, no cost model was found during the systematic literature review. However, there are characteristics present in the studies included in the systematic literature review that could be adopted in the proposed model, which can be considered as a consolidation of the characteristics observed in the studies included in the review and the practice of the researched public hospital while making use of costing methodologies compatible with the objectives of the model.

The first important definition related to patient flow. This study considers all stages ranging from the patient’s admission to their discharge, as presented in Figure 2 which shows the surgical hospitalization process. This wider approach is aligned with all studies reviewed [30,31,32,33,34,35,36,37,38,39,40,41,42,43,44], reflecting the total cost of the surgical procedure from the hospital perspective and differing from some excluded studies whose scope was only the costs in the operating room.

Regarding the costing method, as seen in Table 3, it was found that some studies did not declare which method was adopted [30,32,35,38,39,43]. On the other hand, some research investigates costs for a limited number of surgeries using bottom-up methods [31,33,34,36,37,40,41,42,44]. In all these cases, no more than three procedures were assessed due to the costing method being more complex and requiring a longer time to be carried out, which is a limitation if there is a need for a major number of surgery hospitalization costs, e.g., for decision making about a portfolio of multiple different surgeries. This study adopts the micro-costing top-down method. This method was chosen because it allows for balance between the accuracy of the information and the feasibility of the cost estimate [28,47] and fills the gap related to assessing a vast number of different surgeries in the same hospital.

Department costs, with the definition of cost centers such as the UICC, ICU, and exams, is coherent with the micro-costing top-down method adopted [27,28] and was also observed in reviewed studies, as can be seen in Table 4 which lists the cost centers mentioned in the included studies and the resources considered. Scales et al. [30] and McCarthy et al. [38] separated the information in surgical costs and costs of hospitalization. Ramiarina et al. [31] adopted cost centers and classified them in three categories: an expense-generator center, support and administration services, and auxiliary diagnostic and treatment services. Dowsey et al. [34] present their results by cost category and Vogl et al. [41] define cost centers and calculate average costs for each center.

To allocate costs to departments or directly to procedures, the distinction in direct and indirect costs adopted in this study was also used by Handy et al. [33], Kamath et al. [36], Monsivais et al. [43], and Wise et al. [44]. After classifying and separating the costs, it was observed in some studies that the cost of the patient’s daily stay was defined [30,31,32,38]. This daily cost multiplied by average LOS results in the average cost of hospitalization. Kurich et al. [37], McDonald et al. [38], and Wise et al. [44] found that LOS has a significant impact on the cost of surgical hospitalization. This cost driver used in the proposed model was also considered in several studies regarding surgery costs [30,31,32,33,37,38,41,43,44].

The use of central tendency measures was used in this study for the application of cost drivers, such as the average LOS, median costs of supplies and medicines, and also for the presentation of results. The adoption of these measures was observed in other studies [31,32,38,39,40,43] and is appropriated due to the intrinsic variability of each surgery, which may be different for each patient.

Another pattern observed in other studies is the use of data extracted from computerized systems [31,33,34,36,37,41,42,43,44]. This process makes access to the necessary data more feasible and allows it to be carried out more quickly. The use of computerized data sources proposed in this study is necessary to achieve the objective of assessing a vast number of different surgeries.

Regarding personnel costs, many differences were found among studies. Scales et al. [30] and Kohan et al. [32] established them based on the national MEDICARE reimbursement table, thus the cost was not obtained from the studied hospital. Handy et al. [33] and McCarthy et al. [39] did not include them in their estimates. Kurichi et al. [37] did not show whether personnel costs (e.g., doctors) were considered. In this study, Personnel cost is calculated by department but is segregated from the Operating room cost and the Hospitalization cost. Thus, this cost can be treated independently and allow comparability between institutions with different hiring models or studies without this information.

As observed, there is no universally accepted model for determining costs of surgical admissions. There is a wide variation in the methodology applied in studies with a similar purpose and most cost studies covered only one or a few procedures, which demonstrates the difficulty in carrying out such studies. These studies were usually dependent on an investigation full of manual data collection procedures, which also makes them difficult to repeat.

## 5. Conclusions

The modeling process made it possible to identify the flow of the surgical patient within a hospital. This step is essential int identifying cost sources and to understand that the cost generated occurs from admission to discharge.

The micro-costing top-down method was adopted to develop the cost model allowing for a balance between the accuracy of the information and the feasibility of the cost estimate. Thus, the proposed model fills two gaps in the literature which gives originality to this research. The first gap is filled with the standardization of a model for calculating the costs of surgical hospitalizations. The second gap is that the proposed model is applied to assess a wide number of different surgeries in the same hospital, while most research investigates costs for a limited number of surgeries.

The studies included in the systematic literature review contributed to the definition of essential characteristics of the proposed model, i.e., defining the scope of patient flow; the use of cost centers and treating direct and indirect costs; the adoption of central tendency measures in calculations and the presentation of results; the support of computerized systems for the extraction and processing of data; and determining how to define a specific approach to personnel costs. These features make up the designed model which consists of three distinct cost blocks: the “Operating room cost”, the “Hospitalization cost”, and the “Personnel cost”. These blocks allow hospital managers to observe their costs in greater detail, which also allows for comparisons between different hospitals and studies.

Flexibility stands out as an essential advantage of the proposed model, as its application is possible for calculating costs in different areas and contexts of hospitalization which encompasses elective and urgent surgeries of medium and high complexity performed in public hospitals and in private hospitals. The proposed model can also be adapted to other hospitalization types, such as clinical hospitalization, pediatric hospitalization, and obstetric hospitalization. This allows for application in other health establishments that are general or specialized in some of these types of hospitalization, which expands its potential.

The limitations of this research are the number of databases consulted, the search parameters for the literature review (that may have restricted the results), and the scope of the study which limited it to modeling the problem and not presenting values of its application.

For future studies, this cost model will be applied in the public teaching hospital analyzed and has been suggested to be applied in other hospitals. Studies aimed at analyzing KPIs to evaluate the model are also suggested, as well as indicators derived from its application, such as “Average cost of a portfolio of surgical procedures performed by the Hospital” and “Index of financial sustainability of the surgical procedure”. The proposed cost model could provide information with a high degree of accuracy and celerity, which can induce better decision making. This becomes more relevant in public health, especially in low- and middle-income countries, which faces a lack of resources and whose positive effects can improve healthcare and provide better functioning of the health system.

## Figures and Tables

**Table 1:** Justifications for excluding the full texts.

Reason	Quantity
Performs an estimate based on the conversion rate available in a database	36
Reports that hospitalization costs were obtained from the institution without presenting a costing method	7
Reports that an external database was consulted without presenting a costing method	7
Does not analyze the cost of surgical hospitalization	4
Not available for download	4
Focus on costs such as clinical treatment, diagnostics, and/or outpatient procedures	2
Values presented refer to hospital billing data to the health system instead of costs	2
Considers the amounts paid to the hospital instead of their cost	1
Focuses on a literature review of cost-effectiveness studies that do not address cost analysis models	1
Values are a combination of costs and fees recorded in electronic medical records	1
Total	65

**Table 2:** Bibliometric data.

Year	Author	References	Title	Study Country	Journal
2005	Scales Jr., C.D., Jones, P.J., Eisenstein, E.L., et al.	[30]	Local cost structures and the economics of robot assisted radical prostatectomy	United States	*Journal of Urology*
2008	Ramiarina, R., Almeida, R.M.V.R., Pereira, W.C.A.	[31]	Hospital costs estimation and prediction as a function of patient and admission characteristics	Brazil	*The International Journal of Health Planning and Management*
2010	Kohan, E., Hazany, S., Roostaeian, J., Allam, K., et al.	[32]	Economic advantages to a distraction decision tree model for management of neonatal upper airway obstruction	United States	*Plastic and Reconstructive Surgery*
2011	Handy Jr., J.R., Denniston, K., Grunkemeier, G.L., et al.	[33]	What is the inpatient cost of hospital complications or death after lobectomy or pneumonectomy?	United States	*Annals of Thoracic Surgery*
2011	Dowsey, M.M., Liew, D., Choong, P.F.M.	[34]	Economic burden of obesity in primary total knee arthroplasty	Australia	*Arthritis Care and Research*
2011	Vanni A.J, Stoffel J.T.	[35]	Ileovesicostomy for the neurogenic bladder patient: outcome and cost comparison of open and robotic assisted techniques	United States	*Urology*
2012	Kamath, A.S., Sarrazin, M.V., Vander Weg, M.W., et al.	[36]	Hospital costs associated with smoking in veterans undergoing general surgery	United States	*Journal of the American College of Surgeons*
2013	Kurichi, J.E., Vogel, W.B., Kwong, P.L., et al.	[37]	Factors associated with total inpatient costs and LOS during surgical hospitalization among veterans who underwent lower extremity amputation	United States	*American Journal of Physical Medicine and Rehabilitation*
2014	McDonald, M.R., Sathiyakumar, V., Apfeld, J.C., et al.	[38]	Predictive factors of hospital LOS in patients with operatively treated ankle fractures	United States	*Journal of Orthopaedics and Traumatology*
2014	McCarthy, I.M., Hostin, R.A., Ames, C.P., et al.	[39]	Total hospital costs of surgical treatment for adult spinal deformity: An extended follow-up study	United States	*Spine Journal*
2015	Sözmen, K., Pekel, Ö., Yılmaz, T.S., et al.	[40]	Determinants of inpatient costs of angina pectoris, myocardial infarction, and heart failure in a university hospital setting in Turkey	Turkey	*Anadolu Kardiyoloji Dergisi*
2016	Vogl M, Warnecke G, Haverich A, et al.	[41]	Lung transplantation in the spotlight: Reasons for high-cost procedures	Germany	*Journal of Heart and Lung Transplantation*
2018	Menendez, M.E., Lawler, S.M., Shaker, J., et al.	[42]	Time-driven activity-based costing to identify patients incurring high inpatient cost for total shoulder arthroplasty	United States	*Journal of Bone and Joint Surgery-American Volume*
2019	Monsivais, D; Morales, M; Day, A; et al.	[43]	Cost Analysis of Endovascular Coiling and Surgical Clipping for the Treatment of Ruptured Intracranial Aneurysms	United States	*World Neurosurgery*
2020	Wise K, Blaschke BL, Parikh HR, et al.	[44]	Variation of the Inpatient Cost of Care in the Treatment of Isolated Geriatric Intertrochanteric Hip Fractures	United States	*Geriatric Orthopaedic Surgery and Rehabilitation*

Abbreviation: LOS, Length of Stay.

**Table 3:** Qualitative analysis of the articles included in the study.

Author	References	Proposed Cost Calculation Model?	Is There a Declared Costing Methodology? Which One?	Study Objective	Number of Different Surgical Procedures
Scales Jr., C.D., Jones, P.J., Eisenstein, E.L., et al.	[30]	No	No	Compare costs between procedures with different techniques	2
Ramiarina, R., Almeida, R.M.V.R., Pereira, W.C.A.	[31]	No	Yes, unit cost	Estimate cost per specialty/clinic and propose a model to analyze the relationship between costs and patient admission characteristics	-
Kohan, E., Hazany, S., Roostaeian, J., Allam, K., et al.	[32]	No	No	Ascertain the economic advantages of an alternative treatment model compared to conventional treatment	2
Handy Jr., J.R., Denniston, K., Grunkemeier, G.L., et al.	[33]	No	Yes, microallocation	Understand the cost of complications in patients who have undergone thoracic surgery	2
Dowsey, M.M., Liew, D., Choong, P.F.M.	[34]	No	Yes, bottom-up	Estimate obesity-related overhead associated with knee arthroplasty	1
Vanni A.J, Stoffel J.T.	[35]	No	No	Compare costs between procedures with different techniques (open and robotic)	1
Kamath, A.S., Sarrazin, M.V., Vander Weg, M.W., et al.	[36]	No	Yes, ABC	Compare costs of surgical hospitalizations between smoking and non-smoking patients	-
Kurichi, J.E., Vogel, W.B., Kwong, P.L., et al.	[37]	No	Yes, ABC	Investigate factors associated with cost and LOS	1
McDonald, M.R., Sathiyakumar, V., Apfeld, J.C., et al.	[38]	No	No	Relate anesthetic assessment score to LOS and costs	1
McCarthy, I.M., Hostin, R.A., Ames, C.P., et al.	[39]	No	No	Calculate specific procedure cost	1
Sözmen, K., Pekel, Ö., Yılmaz, T.S., et al.	[40]	No	Yes, bottom-up	Determine cost impact of factors related to cardiovascular diseases	3
Vogl M, Warnecke G, Haverich A, et al.	[41]	No	Yes, activity-based micro-costing	Calculate specific procedure cost	1
Menendez, M.E., Lawler, S.M., Shaker, J., et al.	[42]	No	Yes, TDABC	Calculate specific procedure cost	1
Monsivais, D; Morales, M; Day, A; et al.	[43]	No	No	Compare costs between procedures with different techniques	2
Wise K, Blaschke BL, Parikh HR, et al.	[44]	No	Yes, ABC	Identify variables that can impact the cost of surgery	1

Abbreviations: LOS, Length of Stay; ABC, Activity-Based Cost; TDABC, Time-Driven Activity-Based Cost.

**Table 4:** Types of costs mentioned in the articles included in the review.

Author	References	Cost Centers	Resources
Scales Jr., C.D., Jones, P.J., Eisenstein, E.L., et al.	[30]	Surgical costs	Operating room, equipment, robot cost/case, anesthesia technical cost, post-anesthesia care, professional fees (surgeon and anesthesia)
		Nonsurgical costs	Hospital room/board (feeding), pharmacy/transfusion/laboratory
Ramiarina, R., Almeida, R.M.V.R., Pereira, W.C.A.	[31]	Expense-generator centers	Surgery clinics and their support services, cardiology, clinical medicine and neurology clinics, intensive care unit, consultation, exams, hemodialysis treatment directly concerning external patients
		Support and administration services	Hospital supervision and control, information services
		Auxiliary diagnostic and treatment services	Blood bank, endoscopy, hemodialysis, laboratories, chemotherapy, imaging
Kohan, E., Hazany, S., Roostaeian, J., Allam, K., et al.	[32]	Not available	Operating room facility fees, anesthesia fees, equipment costs, itemized costs for routine preoperative and postoperative laboratory tests, medicines associated with each patient’s operation
Handy Jr., J.R., Denniston, K., Grunkemeier, G.L., et al.	[33]	Anesthesia	Not available
		Surgical services	Not available
		Infusion/support	Blood bank, intravenous therapy, nutrition services, supply, distribution
		Diagnostics	Imaging, endoscopy, electrodiagnostics
		Housing	Preoperative, post-anesthesia care unit, intensive care unit, medical-surgical units
		Laboratory	Clinical, serology, reference testing
		Therapy	Respiratory, speech, physical, occupational, oncology
		Pathology	Not available
		Pharmacy	Not available
		Emergency	Emergency physicians and services
		Other	Wound care, hemodialysis
Dowsey, M.M., Liew, D., Choong, P.F.M.	[34]	Not available	Medical (surgical and nonsurgical), nursing, allied health, imaging, pathology, pharmacy, operating room (includes implant costs)
Vanni A.J, Stoffel J.T.	[35]	Not available	Room, board (feeding), operating room fees, surgical supplies, surgeon and anesthesiologist professional procedural charges, recovery room/intensive care unit costs, robotic maintenance fees
Kamath, A.S., Sarrazin, M.V., Vander Weg, M.W., et al.	[36]	Not available	Labor, supplies, equipment, laboratory tests, X-rays, nursing hours, security, administration
Kurichi, J.E., Vogel, W.B., Kwong, P.L., et al.	[37]	Not available	Surgery, radiology, nursing, laboratory, pharmacy, other unspecified costs
McDonald, M.R., Sathiyakumar, V., Apfeld, J.C., et al.	[38]	Not available	Not available
McCarthy, I.M., Hostin, R.A., Ames, C.P., et al.	[39]	Not available	Supply costs, operating room
Sözmen, K., Pekel, Ö., Yılmaz, T.S., et al.	[40]	Not available	Diagnostic procedures costs, medical supply costs, laboratory tests, interventional treatment costs, surgery, ward cost, physiotherapy, physicians’ costs, nursing costs
Vogl M, Warnecke G, Haverich A, et al.	[41]	Ward, intensive care, operating room and anesthesia, diagnostics and therapy, laboratories	Labor (physicians, nursing, technical staff), drugs, materials (expendables), infrastructural costs (technical and management)
Menendez, M.E., Lawler, S.M., Shaker, J., et al.	[42]	Not available	Implant, medications, operating room consumables, personnel cost (preoperative through operating room and post-anesthesia care unit through discharge)
Monsivais, D; Morales, M; Day, A; et al.	[43]	Not available	Room costs for intensive care unit and wards (includes physician and nursing charges), operating room costs, respiratory care, medical supplies, medications, laboratory tests, imaging, surgery staff costs (surgeon, anesthesia, and nursing), physical therapy, occupational therapy, speech therapy, cardiology services, respiratory therapy, emergency services, overhead costs (electrical power, running water, janitorial, maintenance services)
Wise K, Blaschke BL, Parikh HR, et al.	[44]	Not available	Surgical implants, inpatient postoperative rehabilitation, surgical costs, nursing, respiratory therapy, pharmacy, patient labs, emergency care, diagnostic imaging, hematology, cardiology, critical care, administration, information technologies support, human resources

**Table 5:** Relationship between cost blocks, cost centers, and cost drivers.

Cost Blocks	Cost Centers	Cost Drivers
Operating room Cost	Operating room	Frequency of surgical hospitalizations (number of procedures performed)
Hospital Cost	UICC	Number of hospital days at the UICC
		Length of stay at the UICC for each procedure
	ICU	Number of hospital days at the ICU
		Length of stay at the ICU for each procedure
	Exams	Proportion of exams for surgical hospitalizations compared to the total
	NIR	Proportion of surgical hospitalizations compared to the total
Personnel Cost	Operating room	Personnel cost per surgery
		Frequency of surgical hospitalizations (number of procedures performed)
		Proportion of surgeries by each operating room
	UICC	Personnel cost for each day at the UICC
		Number of hospital days at the UICC
		Length of stay at the UICC for each procedure
	ICU	Personnel cost for each day at the ICU
		Number of hospital days at the ICU
		Length of stay at the ICU for each procedure
	Exams	Personnel cost per exam
		Proportion of exams for surgical hospitalizations compared to the total
	NIR	Personnel cost per admission
		Proportion of surgical hospitalizations compared to the total

Abbreviations: UICC, Inpatient Unit of the Surgical Clinic; ICU, Intensive Care Unit; NIR, Internal Regulation Center.

## Data Availability

The data presented in this study are available on request from the corresponding author.
